# GFAP and S100B: What You Always Wanted to Know and Never Dared to Ask

**DOI:** 10.3389/fneur.2022.835597

**Published:** 2022-03-21

**Authors:** Damir Janigro, Stefania Mondello, Jussi P. Posti, Johan Unden

**Affiliations:** ^1^Department of Physiology and Biophysics, Case Western Reserve University, Cleveland, OH, United States; ^2^FloTBI, Cleveland, OH, United States; ^3^Department of Biomedical and Dental Sciences and Morphofunctional Imaging, University of Messina, Messina, Italy; ^4^Department of Neurosurgery, Neurocenter, Turku Brain Injury Center, Turku University Hospital, University of Turku, Turku, Finland; ^5^Department of Operation and Intensive Care, Hallands Hospital Halmstad, Lund University, Lund, Sweden

**Keywords:** blood-brain barrier, neurodiagnostics, astrocytes, brain damage, brain hemorrhage, blood biomarkers, point-of-care, kinetics

## Abstract

Traumatic brain injury (TBI) is a major global health issue, with outcomes spanning from intracranial bleeding, debilitating sequelae, and invalidity with consequences for individuals, families, and healthcare systems. Early diagnosis of TBI by testing peripheral fluids such as blood or saliva has been the focus of many research efforts, leading to FDA approval for a bench-top assay for blood GFAP and UCH-L1 and a plasma point-of-care test for GFAP. The biomarker S100B has been included in clinical guidelines for mTBI (mTBI) in Europe. Despite these successes, several unresolved issues have been recognized, including the robustness of prior data, the presence of biomarkers in tissues beyond the central nervous system, and the time course of biomarkers in peripheral body fluids. In this review article, we present some of these issues and provide a viewpoint derived from an analysis of existing literature. We focus on two astrocytic proteins, S100B and GFAP, the most commonly employed biomarkers used in mTBI. We also offer recommendations that may translate into a broader acceptance of these clinical tools.

## Introduction

Over the past 20 years, there has been unprecedented progress in the development and availability of blood- or peripheral fluid-based brain injury biomarkers to improve the diagnosis and clinical characterization of patients with neurological disorders, offering also remarkable opportunities toward the understanding of disease pathophysiology and influencing medical decision-making and therapeutic strategies. Traditionally, research into brain diseases, particularly research related to traumatic brain injury (TBI), has focused on neuronal damage. In fact, “brain damage” has often been used as a synonym for neuronal cell death (1). Thus, it is to some extent surprising that the *astrocytic* proteins S100B and glial fibrillary acidic protein (GFAP) are among the most studied and promising peripheral biomarkers (2). Their elevations in peripheral body fluids in a wide range of neurological and psychiatric conditions have been ascribed to ongoing brain injury or dysfunction (3), increased blood-brain barrier (BBB) permeability (1, 4, 5), or both. In addition, they are being used to diagnose TBI in research studies and clinical settings (2, 6–8). In this work, we review and compare S100B and GFAP's pathobiological characteristics and discuss the evidence for their use in different neurological conditions (9–12) with a focus on mild traumatic brain injury (mTBI). We also provide practical recommendations for their validation and implementation in clinical settings, considering the analytical aspects and outlining limitations and knowledge gaps that need to be addressed in future studies.

Notably, S100B and GFAP have distinct characteristics and kinetic patterns, and have been show to yield independent and complementary information. Hence, they can be synergistically adopted in clinical decision making. Refinement of disease phenotype and outcome prediction may also benefit from their combined use.

## Properties of S100B and GFAP

**Figure 1:**
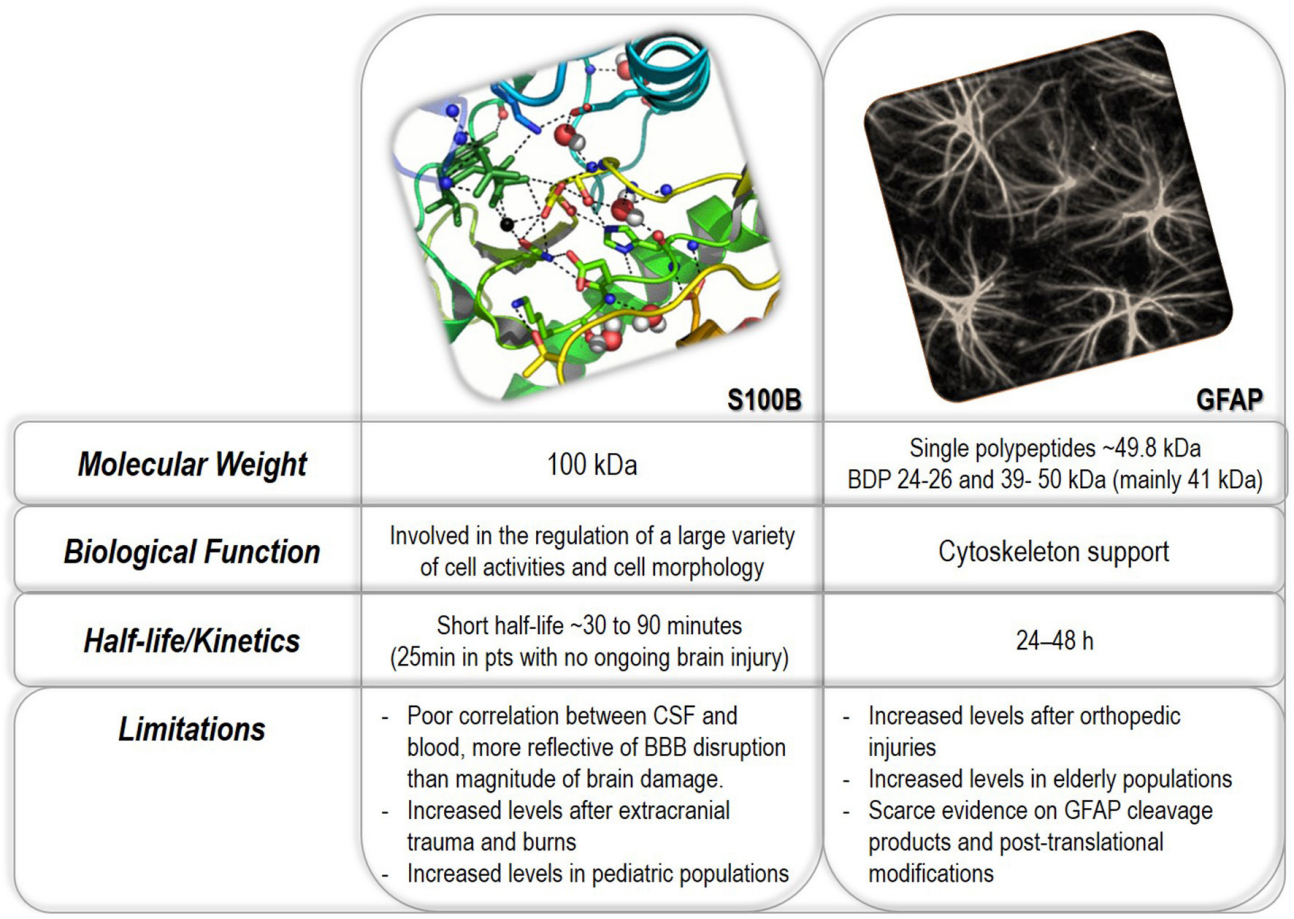
See text.

Figure 1 summarizes the properties of S100B and GFAP. S100B is a small homodimeric protein consisting of two β subunits, with a molecular weight of ~21 kDa (13). It belongs to a multigenic family of Ca2+-binding proteins (i.e., regulators of intracellular levels of calcium) involved in a variety of intracellular and extracellular activities, including neuronal differentiation, survival and proliferation, protein phosphorylation, and cell motility (13, 14). Besides executing intracellular functions under physiological or pathological conditions, S100B is also actively secreted by astrocytes and adipocytes into the extracellular fluid, where, in particular in the brain, it seems to play an important role in tissue development and repair (15, 16). S100B released by adipocytes may (17) or not (18) influence peripheral levels. Recent evidence has shown that S100B is responsible for maintaining neuronal gamma rhythms in the hippocampus (19, 20).

Organ S100B expression at the mRNA level is limited to astrocytes, leukocytes, melanocytes the testis (21). See also https://www.proteinatlas.org/ENSG00000160307-S100B/tissue. S100B *protein* is present in adipocytes, striated muscle, enteric glial cells, adipocytes, chondrocytes, melanocytes, and heart muscle [e.g., see (21)]. Still, the highest protein concentrations have been detected in astroglial cells (22). The presence in extracranial organs is due to the cellular uptake of circulating S100B (21) by a mechanism recently proposed in ref. (23) showing that clathrin and lipid rafts contribute to the internalization of S100B. The contribution of expression outside the CNS to the blood signal in a healthy individual is minor as the highest value, including all extracranial sources, is well below the levels seen in TBI (24). However, the current orthodoxy is that the clinical use of S100B in mTBI should be limited to patients without major non-brain injuries (25, 26) though extracranially released S100B is quickly eliminated (27). Of note, a recent large-scale multicenter study found no effects of multi-trauma on S100B levels (28).

Glial fibrillary acidic protein (GFAP) is a cytoskeletal monomeric filament protein present in astroglial cells located in white and gray matter (29). GFAP protein is also present in non-glial and non-CNS cells, such as non-myelinating Schwann cells, chondrocytes, testicular Leydig cells, enteric glia, podocytes, mesangial cells, and liver and pancreas, stellate cells (30–34). GFAP is released into the bloodstream both as an intact protein (50 kDa) and as breakdown products (18–44 kDa) derived from calpain- and caspase-cleavage, in particular caspase 3, 6, and 9 (35, 36).

Glia-derived proteins present in normal cerebrospinal and interstitial brain fluids (CSF and IF, respectively) may act as indicators of BBB damage (BBBD) when measured in peripheral body fluids (5, 24). Their increase may be simply due to a passive, rapid distribution across disrupted endothelial tight junctions or by cellular damage to glial cells after a traumatic event (14, 37–39). The fact that the BBB prevents S100B from leaving the brain was experimentally demonstrated by showing that CSF increases do not result in measurable serum changes unless an event disrupting the BBB, such as mTBI, was superimposed (5). Comparable evidence for GFAP is lacking, but a quantitative model of GFAP and S100B transfer process across a leaky BBB has been developed (24). The half-life of S100B was shorter than GFAP, mostly due to different kidney filtration rates of protein with different molecular weights.

An alternative to direct passage across a disrupted BBB was recently proposed (40). The so-called glymphatic system (41, 42) is, according to this hypothesis, responsible for the migration of S100B and GFAP from the injured brain into the peripheral blood. There are several important considerations that glymphatic drainage of astrocytic protein implies. First, it may explain secondary delayed biomarker surges after TBI (38, 39). Cerebral edema may trigger pathological changes in GFAP and S100B brain synthesis, resulting in more significant extravasation via the glymphatic pathway. Second, it may also explain the accumulation of tau protein in the CNS after severe trauma (43). However, the glymphatic drainage hypothesis does not fit with the kinetic data of glial markers acute appearance in the blood (1–2 h vs. ~20), nor does it seem to apply to mTBI where frank brain lesions are seldom observed.

Following brain injury, S100B can also be passively released by dying damaged astrocytes into the circulation via the BBB, with rapid clearance thereafter (half-life 60–120 min). This may be the main trigger of secondary, delayed increases in S100B (or GFAP) after TBI (38, 39). These are usually correlated with secondary adverse events.

## What Do Elevations in S100B or GFAP Represent?

**Figure 2:**
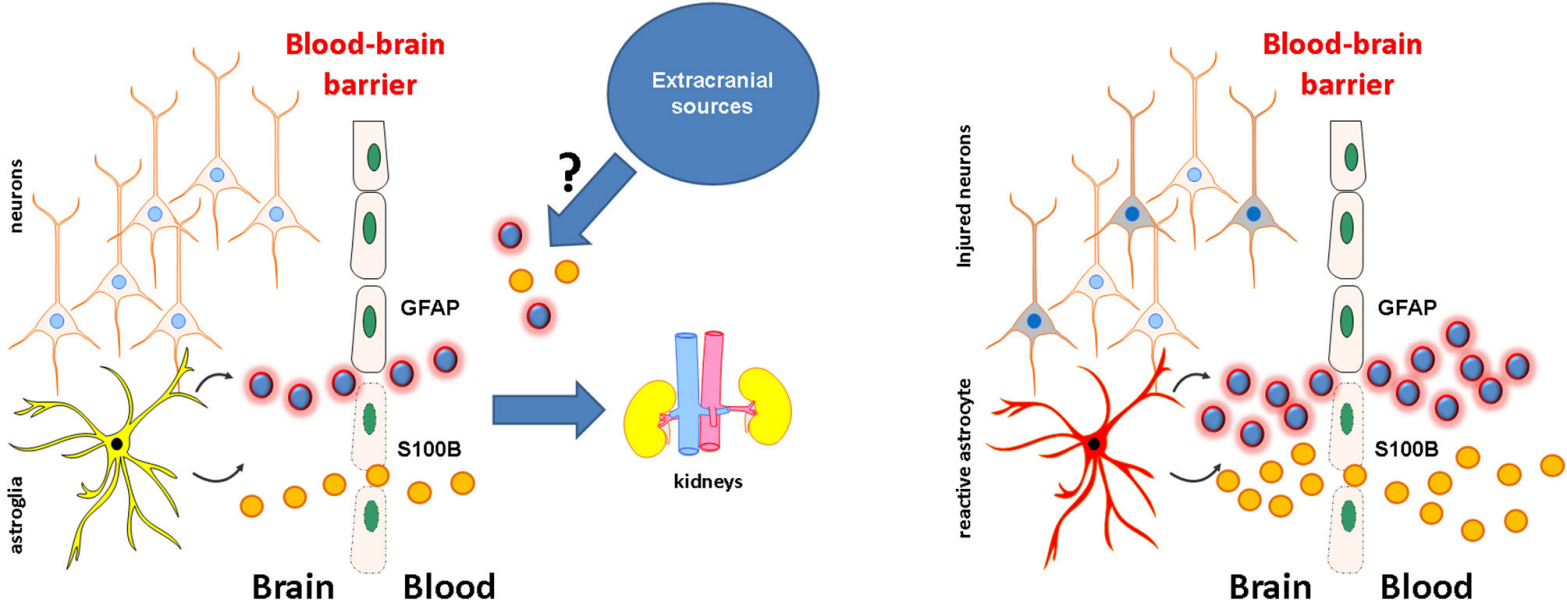
See text.

Figure 2 describes in a graphical format the following paragraphs. Since BBB “leakage” is a hallmark of many neurological diseases (44, 45) or even subclinical events such as subconcussive head hits (46, 47), increases in peripheral levels of glial proteins occur in various brain diseases. In the presence of an anatomical lesion or noxious event (e.g., a seizure or TBI), *ex novo* synthesis by reactive astrocytes may further elevate IF and CSF levels, which will increase their peripheral levels across a permissive BBB. Thus, a biomarker “dose-response” related to damage severity would be significant; none of todays' markers consistently gives a linear relationship between levels and parenchymal lesion size/severity.

The issue of whether or not a disrupted BBB in the absence of brain damage is sufficient to elevate serum levels is not purely academic. Within the spectrum of TBI, a subconcussive injury group was identified by a quantitative dynamic contrast MRI protocol to show that American football athletes experience BBB disruptions even when no concussion is present (48). BBB damage was also reflected by elevated blood S100B in non-concussed football players with high head impact scores reported in two independent studies (46, 47). This raises the intriguing possibility that in mTBI clinical trials (see below), false-positive elevations of GFAP and S100B in the absence of demonstrable parenchymal involvement may reflect BBB damage not visible on CT imaging.

While most of the available clinical findings relate to TBI and its radiological sequelae, other studies focused on ischemic and hemorrhagic stroke [S100B and GFAP (49)], brain neoplasms or metastases (S100B) (9, 50) and GFAP (51), infectious diseases with CNS involvement (S100B) (52), or psychiatric disorders (S100B) (53). Therefore, due to the mechanism of S100B (and GFAP) release across a dysfunctional BBB and from damaged parenchymal cells, these biomarkers lack specificity for any given neurological disease when sampled in the periphery. Thus, an elevation of S100B or GFAP cannot be interpreted as a stroke or TBI signal without considering the patient's clinical context. Therefore, the illness's setting and its mode of onset are paramount in translating any peripheral glia-derived biomarker analysis into a medical diagnosis.

A literature review on blood biomarkers for brain diseases reveals that the term “statistically significant increase” is often used to suggest a clinically meaningful effect on biomarker levels. For example, ref. (17) reports an increase of S100B with body mass index (BMI). The correlation between these two variables was statistically significant, yet only a small percentage of S100B levels were above the normal threshold when using the test employed (0.12 ng/ml). Thus, while a correlation between BMI and S100B existed, the clinical significance of the increase may not be significant. Nevertheless, it is important to understand why obesity should influence blood levels of S100B. The most parsimonious explanation is that fat tissue (adipocytes) release S100B and that more fat tissue will release larger quantities. A caveat of this explanation is that in the article mentioned above, a positive relationship between S100B and BMI was true only for values of BMI >30; in other words, the correlation between S100B and BMI was due to increases of S100B in obese individuals. If adipose tissue were a source of venous S100B, one would expect this to hold true also within the normal-to-overweight range. The question thus is, are there any comorbidities of obesity other than BMI that may influence *brain* release of S100B? Hypertension elevates S100B by a mechanism involving the cerebral vasculature (54), and hypertension is a comorbidity of obesity. It is thus possible that the elevated levels of S100B in obese individuals are not due to obesity itself but rather to complications associated with an elevated BMI.

## The Rationale for the Use of S100B and GFAP in Traumatic Brain Injury

TBI is defined as a perturbation of brain function or a pathological brain structure lesion caused by an external force (55). In mTBI (mTBI), the duration of unconsciousness is a few minutes (up to 30 min) and post-traumatic amnesia up to 24 h. However, in many cases of mTBI, patients may not lose consciousness. The best-known classification criteria for mTBI are the American Congress of Rehabilitation Medicine (ACRM) (56) and World Health Organization (WHO) classifications (56). mTBI comprises the vast majority (80–90%) of all TBI cases. However, this figure is considered an underestimation, as a significant proportion of TBI victims do not seek medical attention (57). Even a mTBI may result in complex events, including functional, metabolic, and inflammatory alterations. These changes are reflected in the levels of brain-derived proteins released into the circulation and CSF (58).

TBI is among the most common causes for seeking emergency medical attention. Patients with mTBI are, by definition, conscious when they arrive at emergency departments (57). Considering the high numbers of these patients, the substantial healthcare burden is obvious, independent of whether mTBI victims experienced a short period of unconsciousness and amnesia. From a clinical viewpoint, the most common questions encountered by emergency physicians dealing with head injury patients are related to diagnosing TBI severity, need to undergo a head CT, need for hospitalization, and prognosis of long-term sequelae.

mTBI diagnosis is often challenging in the emergency department setting because patients are typically intact on neurological examination, and criteria for acute head CT are often not met (6, 59, 60). Nevertheless, CT is the gold standard to identify the subgroup of patients with intracranial pathology necessitating in-hospital or neurosurgical care (61). Several international guidelines have been developed to aid in decision-making about how to risk-stratify patients for the need of acute head CT. These guidelines are based on the patient's medical history, medications, initial and evolving symptoms, and findings on neurological examination (62). Regardless, a significant proportion of patients who undergo head CT at admission have a negative scan for macroscopical brain lesions (63). This is clinically problematic as there is an iatrogenic risk for radiation-induced neoplasia associated with CT scanning (64). This risk is especially pronounced in children and infants.

Blood-based biomarkers are widely assessed for estimation of disease severity and progression in many areas of medicine. The most studied indication for clinical blood-based biomarker use in neurotraumatology is stratifying patients for CT imaging after a head injury [e.g., Scandinavian guidelines (6)]. Essentially, the biomarker used in this setting further stratifies a group of intermediate-risk mTBI patients to low-risk, hence omitting the need for CT and/or hospitalization. Studies have confirmed the excellent sensitivity of S100B in this setting, although the specificity is disappointingly low and may hamper effective implementation.

In severe TBI, current management and care involve a combination of neurosurgery and neurointensive care. During this period, dynamic changes in the brain (such as hemorrhagic or ischemic complications) may be challenging to detect and hence treat. A biomarker may add valuable diagnostic information in this setting. Although there is no strict neuroprotective drug in clinical use, many have been proposed and are presently being evaluated. Other neuroprotective measures following TBI may also need to be assessed and followed during the time-course of TBI development. Insights relating to long-term outcomes following TBI may aid clinicians in the management and timing of rehabilitation efforts. Outcome prediction may also be informative regarding the level of care, questions concerning life-support measures, and information to family and relatives.

## Clinical Studies and Current Use in TBI (Including Injuries, CT, MRI, Outcome Variables)

The current management of TBI involves different phases. Immediately after a TBI event, the patient and/or bystanders decide whether to involve health care professionals. Initial symptoms and clinical signs may be worrisome (such as loss of consciousness (LOC), amnesia, seizures, and neurological deficits), which usually lead to a health care contact, even if these symptoms/signs often subside. In some instances, such as in sporting activities, specific informatic questionnaires may be used to manage patients (see below). In any case, a group of these patients will end up seeking medical care, most of them within the first few hours after the event. These cases generally present to Emergency Departments (ED's), although some may be seen in primary care facilities. Irrespective of where the patients seek care, the initial assessment is similar. Elements of patient history, including medications (primarily blood thinners), description of the traumatic event, and initial symptoms, are combined with clinical examination to stratify patients according to the risk of intracranial injury, particularly those that may require intervention (such as neurosurgery). The foundation of this process is a score based on the consciousness level of the patient, in practice, often the Glasgow Coma Scale (GCS), but many other parameters can be included. Based on all these factors, patients are either briefly observed, admitted to the hospital, transferred to another hospital, receive a CT scan (or a combination of these), or discharged without further investigation.

It is notoriously difficult to stratify these patients accurately. International guidelines and decision rules, either based on derivation and validation cohorts or an evidence-based process, simplify the above factors to facilitate management for the treating physician. Despite this approach, many patients still receive unnecessary CT scans and/or hospitalization as the guidelines are designed to maximize sensitivity (negative predictive value) for intracranial complications after TBI, leading to a low specificity (positive predictive value). More importantly, most elements of these guidelines are based upon subjective measures either supplied from the patient (who has suffered a TBI, possibly clouding accurate reporting) or the treating physician (inter-rater agreeability may be low). Additionally, many patients are children, elderly, or may suffer from dementia, and many patients are intoxicated (65). All these factors further complicate clinical judgment and even guideline use.

A biomarker is an objective measure. Results are presented as continuous variables, allowing a cutoff to be established, often based on receiver operator curve (ROC) analysis with a clinically relevant outcome. The chosen cutoff can either maximize the sensitivity or specificity (or both using two separate cutoffs), depending on the intended use of the biomarker. In mTBI, guideline development has generally used positive (pathological) CT scans and an outcome measure. High sensitivity has been targeted. It is noteworthy that there is a commonly used threshold for S100B (depending on the test used but with clinical-grade Roche and Diasorin tests around 0.1–0.12 ng/ml) whereas (see below) there is no predetermined “normal” threshold for GFAP in most articles published.

### Adults

S100B is the only biomarker that has been incorporated in a guideline including clinical covariates. The use of S100B has been recommended as part of the Scandinavian Guidelines for Initial Management of Minimal, Mild and Moderate Head Injuries in Adults (6, 59, 60). S100B (cutoff <0.1 ug/L when measured with Roche Diagnostics system) can be used as part of the guideline algorithm to rule out the need for head CT in patients with isolated mild head injuries with low clinical risk for intracranial bleeding within 6 h from the injury. The use of S100B in the Scandinavian guideline has recently been validated in an external cohort with a sensitivity of 0.94 and specificity of 0.19 (59, 66). The positive and negative predictive values for acute traumatic lesions on head CT were 0.18 and 0.94, respectively. The results also showed that the Scandinavian Guidelines could be safely used in imaging decision-making within 24 h of head injury (66). The Scandinavian guidelines with S100B incorporated reduce CT usage and costs (67). A recent meta-analysis confirms this approach (68). Other studies have shown, however, the presence of peripheral trauma may impact serum values [e.g., (69)].

GFAP is detectable within 1 h following TBI and peaks within 20–24 h with a half-life of 24–48 h (70). Abnormal serum GFAP levels persist for days after the initial injury (58); GFAP can discriminate patients with TBI and orthopedic controls after 30 days (39), but the relevance of this finding in acute TBI is marginal. GFAP levels are affected by extracranial injuries (71), but no specific guidelines exist on the process of patient selection for the diagnostic use of GFAP.

The recent ALERT-TBI study showed that blood tests including GFAP and Ubiquitin C-terminal hydrolase-L1 (UCH-L1) in CT-positive findings yielded better sensitivity and specificity (7) than the Scandinavian guidelines in the recent validation study (59). The superiority of GFAP over S100B was also noted in two studies (72, 73). The FDA recently approved this test to identify patients in whom a head CT is necessary. The study based on which the approval was granted showed that the results were significantly driven by GFAP and not UCH-L1 (7, 70, 74) [see also (75)]. Unlike the Scandinavian guidelines, the FDA-approved test does not consider clinical covariates such as extracranial injuries or other clinical factors predisposing for intracranial hemorrhage. GFAP is currently not incorporated into any clinical guideline.

In contrast to mTBI, severe TBI (sTBI) is associated with high mortality (76). About 30% of patients with sTBI die, and 50% suffer at least moderate disability after 1 year, although some make almost complete recoveries (77). Initial assessment of severity may be misleading, and severity grading may change during the acute injury phase because TBI is a dynamic process with a complex and heterogeneous pathophysiology. Early outcome predictions are also difficult because of the threat of secondary insults.

The diagnosis of sTBI remains a daily task for neurosurgeons, anesthesiologists, and emergency care physicians and is based on clinical and radiological findings (8, 76). Almost all studies have reported that the ability of S100B or GFAP to discriminate between CT-negative and CT-positive patients is significantly better in patients with sTBI than in patients with mTBI. For instance, the so-far most extensive acute diagnostics study reported that GFAP levels upon admission were highly predictive of abnormal CT findings, outperformed other markers, and complemented clinical variables considered in current CT decision rules. The results were more pronounced in moderate and severe TBI. Surprisingly, the correlation between GFAP and S100B was relatively weak (0.57)—even in patients admitted to intensive care units (8). Similar findings were reported in a smaller study that also included S100B and GFAP. The study examined not only sTBI patients but all severity levels, which leads the results to be driven by the severe cases (78).

Blood-based biomarkers have been investigated in the diagnosis of secondary insults and outcome prediction in sTBI. In the acute phase of severe traumatic brain injury, the prognosis is essential for both nearest of kin and treating physicians. It facilitates decision-making and the choice of the appropriate extent and intensity of treatment measures. Both S100B and GFAP are robust predictors of outcome in patients with sTBI. S100B can identify patients with an unfavorable outcome and the development of brain death or mortality after sTBI (79–81). In other studies, GFAP and S100B were strong predictors of unfavorable outcomes and correlated with injury severity (82, 83). Serum GFAP levels were also significantly higher in patients who died or had an unfavorable outcome (84). The most widely used prognostic models—the CRASH and the IMPACT calculators—use variables available at admission, such as initial severity using the GCS score, age, pupillary reactivity, CT findings, major secondary insults, and laboratory findings (85). There is growing interest in adding biomarkers to existing clinical prognostic models to improve predictive reliability.

Recently, Thelin et al. examined the concentrations of six different protein biomarkers in relation to injury severity and outcome in patients with predominantly severe traumatic brain injury (70% of cases) in the first week after injury (86). The combination of GFAP and neurofilament light protein provided the best improvement in performance in predictive outcome models, including IMPACT. A principal components analysis model revealed clustering of neuronal markers tau, Ubiquitin carboxy-terminal hydrolase L1 (UCH-L1), and the astrocytic markers S100B and GFAP. None of the examined markers were significantly correlated with diffuse axonal damage detected by MRI. Levels of S100B and UCH-L1 were associated with the presence of associated extracranial injury (86). Czeiter et al. reported that levels of GFAP improved the performance of the IMPACT calculator in predicting the outcome of patients with sTBI (8). However, in terms of incremental value to imaging findings, in a study examining the additional value of biomarkers to the Helsinki CT Score to predict outcome in CT-positive TBI patients, neither S100B nor GFAP showed significant prognostic improvement (87).

### Children

Considering the increased risk from ionizing radiation and the challenging clinical examination of children, a reliable brain biomarker would be important in managing mTBI in these patients. Although studies seem promising, with similar diagnostic performance to adult studies (88–93), more data is needed before the test can be recommended in guidelines (94). Interestingly, an ongoing interventional study should add considerable data to this field (95).

In children, predicting the outcome of sTBI is more complex than in adults because of the heterogeneity of the developing brain and the limitations of clinical examination. The lesions seen on CT in children with sTBI have low sensitivity in predicting outcomes. Therefore, novel objective methods are needed to improve or even replace clinical and radiological parameters that have been associated with outcomes in children with sTBI. Prognostic biomarker studies in children with sTBI are only a few. As in adults, S100B is the most studied prognostic biomarker in children. S100B can discriminate between moderate to sTBI and controls (96). Its levels are associated with outcomes in multiple studies, including TBIs of different severities (97–99). In a recent study with a small cohort of children with sTBI, levels of GFAP discriminated between controls and mTBI or sTBI (100). In earlier studies, levels of GFAP have correlated with outcomes in children with sTBI (101, 102).

## Confounding Factors

A major difference between the S100B and GFAP literature is that while to distinguish between CT-positive and negative findings, a consistent cutoff has often been used for S100B, in contrast, in the case of GFAP, the cutoff varies depending on the study. In addition, cutoff values for S100B are usually reported *a priori*, while for GFAP these were derived from data analysis. While this appears to be due to the use of different testing methods, it is nevertheless worth noting that, for example, Bazarian et al. used a CT- cutoff (ng/ml) of 0.022 GFAP/327 UCH-L1 (mTBI, assay: Abbott Laboratories) (7), while Papa et al. used 0.03/0.1 (mTBI–moderate TBI, assay: Banyan Biomarkers Inc.) (103). Moreover, Posti et al., reported cutoffs for GFAP from 0.14 to 0.24 (Quanterix Simoa) depending on the initial clinical severity and presence of possible extracranial injuries (78, 101). In most studies dealing with GFAP, there was no predetermined cutoff, and cutoffs were calculated *post facto* to fit the data (2).

Another difference between the two tests (GFAP vs. S100B) is the lower limit of detection (LLOD). For S100B, the LLODs reported by the vendors (chiefly Diasorin and Roche Diagnostics; LLOD 0.02 and 0.005 ng/ml, respectively) were also reported in most publications. In contrast, the LLOD values for GFAP have varied wildly between studies. By using a very sensitive platform, Bogoslovsky et al. reported a LLOD of 0.0008 (ng/ml), others report a LLOD of 0.1 (28, 74), 0.01 (104, 105), 0.02 (103), 0.008 (70). This, of course, compounds the interpretation of negative predictive values and false negatives. For example, the study by Welch mentioned above (105) (LLOD = 0.01 ng/ml for GFAP) found a substantial number of samples below the LLOD in both CT- and CT+ patients (64 and 21% of all patients, respectively); false negative values in the CT+ group were factored as the value of LLOD, not as false negatives. The multicenter TRACK-TBI effort reported 26% of samples below LLOD for GFAP, but the study did not distinguish those in CT- and CT+; the impact of >¼ of samples below LLOD on NPV was not discussed (74). In another study (103), samples below LLOD were equalized to ½ of LLOD; the percentage of these values in CT+ subjects was not reported.

The kinetic behaviors of serum GFAP and S100B have been investigated (39, 70, 105, 106). In addition, a computer simulation reported acute, transient values for GFAP and S100B after simulated BBBD (24). On average, for S100B in severe TBI, most studies indicated a *t*1/2 of about 24 h, even if very early sampling in these patients reveals rapid decreases (1–2 h). Another study has shown that the elimination of S100B after cardiac surgery is faster and not affected by a moderate decrease in GFR (107). The half-life of S100B has been shown to depend on kidney glomerular filtration (24). The protein GFAP (*n* = 18) appears to have *t*1/2 of about 24–48 h in severe TBI. Papa et al. (70) report elevated levels of GFAP at time = 0, which corresponded to values at admission (within 4 h after TBI). In patients with TBI, 11.6% of samples were below LLOD; in the CT+ group, the low end of the range was the LLOD (0.008 ng/ml). Thus, even when using an ultrasensitive test, some patients with positive intracranial findings present with GFAP levels at or around LLOD. How these values were analyzed when estimating the half-life for GFAP was not discussed.

The fact that according to most studies the kinetic decay of biomarker's occurs within the recommended time window for testing suggests that time of testing should be either standardized (very difficult since TBI diagnosis is not easy to synchronize across different centers) or that time of testing should be included in the determination of a diagnosis. An alternative approach may consist of testing at two-time points, separated by an interval consistent with the kinetics of S100B or GFAP in blood. In the acute diagnosis of TBI (perhaps excluding the need for head CT imaging), longitudinal sampling—at least two samples—may thus be required to assess the trend in biomarker concentration to acquire clinically useful information. Finnish researchers obtained promising results for GFAP in longitudinal measurement in acute diagnosis of stroke (108).

Given that the brain is the primary source of circulating S100B and GFAP, why are their half-life values so different? Several hypotheses can be formulated, including the effect of GFAP and S100B distribution in tissues and the impact of glomerular filtration (GFR). Experimental work in rats demonstrated that blood S100B partitions with tissues where it is taken up primarily by immune-related cells (dendritic cells in the skin, CD4+ cells in the spleen, etc.) (21). Overall, except for skin cells, S100B blood levels are independent of extracranial sources (18, 24). Both GFAP and S100B are found in testes, but this seems to be due to local production rather than uptake. In addition, diffusion from blood to testes is prevented by testicular barrier cells. While the fate of circulating S100B has been studied, to our knowledge, nothing is known about GFAP uptake by peripheral tissues.

The kinetic process of protein excretion depends on molecular size, among other variables. Molecules smaller than 15 kDa pass into urine through glomerular filtration, whereas the kidney can also filter a selected few proteins with molecular weights between 16 and 69 kDa (24). For example, a common excreted protein, cystatin, has a molecular weight only slightly greater than that of S100B 98. It is thus predicted that S100B, owing to its lower molecular weight, will filter faster than its larger GFAP counterpart. This could, in a computer model (24), explain the different half-lives of GFAP and S100B.

The extracranial contribution to peripheral blood levels has been shown primarily for S100B but also for GFAP and UCH-L1 (71, 72, 109, 110). The general understanding of this problem is that the biomarker's presence in non-CNS tissue contributes to the signal measured in blood. This is clearly a confounding factor since, at least in the case of TBI, fractures and tissue damage may occur together with injury to the head. The mechanism of extracranial sources' contribution to blood levels may be the damage of cells expressing S100B or GFAP and subsequent release of cytosolic content in body fluids. This mechanism is assumed correct even though alternative explanations can be provided. For example, the immunodetection system used may allow for cross-reactivity with inflammatory mediators released by peripheral damage. This was confirmed for S100B measured during open-heart injuries (109). An alternative reason for the increased biomarker levels after multi-trauma is the effect of trauma itself on the BBB (111). Multi trauma promotes a pro-inflammatory cascade and broad changes in blood pressure, which may indirectly cause increased permeability of the BBB. In addition, there is accumulating evidence that inflammatory pain states produce significant changes in the BBB permeability (112, 113). Thus, a combination of cellular (activated leukocytes) and molecular (inflammatory mediators) can synergistically upset the dynamic equilibrium which characterizes BBB function.

## Sports, Biomarkers, and TBI

A recent set of review articles (114, 115) has summarized the state of the art in biomarkers' use in sports concussion and TBI. In our review, we wish to underscore a number of factors that are relevant for sport assessment of mTBI and concussions.

Mounting research in the field of sports concussion biomarkers underscores the deleterious effects of brain injury from recreational activity and professional sports. This increased awareness derives perhaps from the concussion liability trial against the USA National Football League and the literature linking chronic traumatic encephalopathy (CTE) to sport-related repetitive concussions. Without going into the merit of the proposed link with CTE [see (116)], concussions in sports deserve a diagnostic approach that is slightly different form the usual approach to TBI. First, the athletes involved are typically young and healthy; in high school sports, concussed athletes may not yet have a fully developed brain. In addition, the diagnosis at the site of injury depends critically on the presence of medical expertise, usually provided by trainers, “soccer moms” or physician-parents. This is an ideal scenario for an objective test to rule out concussion sequelae. The test, however, has to be done in absence of a trained phlebotomist, which is a great opportunity for salivary tests. Several studies have shown the utility of S100B in this context, including the salivary test recently developed by one of us [DJ (115, 117)].

The issue of mTBI/concussion in sports, unlike civilian TBI but in synchrony with military blast injuries, is the repetitive nature of the event. It is not uncommon for an American football player, a soldier, or a boxer, to experience several mTBI episodes. Thus, each acute event should be clinically gauged as a possible chronic disease. A test for the sequelae of repeated TBI is lacking, but the use of autoantibodies against the S100B biomarker itself has shown early promise (46, 118). Autoantibodies against GFAP have also been described (119, 120).

A few reports have shed doubt on the utility of S100B in sports concussion owing to its increases in the absence of head impacts (121, 122). Others have shown that “running the game” does not impact the significance of S100B elevations after a game (46, 47, 118, 123–125). The main point of contentions seems to be whether S100B can be released from extracranial sources and if this will impact the predictive value of the test. Or, in other words, does physical exercise impact the BBB? Normal levels of exercise improve BBB function (126), while strenuous, prolonged super physiological activity impairs it (127). The latter is due to free radical formation. It is thus possible that the increases in S100B after strenuous exercise are due to free radical formation, BBB disruption and elevation in brain-derived S100B (128).

## Conclusions and Future Aspects

A lot of effort has been made to retool the clinical armamentarium used to diagnose TBI. For several reasons, blood biomarkers have become focus of intense research and development. The reasons for focusing on peripheral biomarkers have been discussed in this review. The combined outcome of these endeavors has produced a sizeable number of articles, reviews and reports. Owing to analytical heterogeneity among laboratories, a direct comparison across studies is not always possible. This is in particular true for GFAP where a broad range of thresholds and LOD have been published. Future side-by-side studies need to use predetermined cutoff values and reproducible, publicly available, measurement strategies.

With the advent of POC plasma testing solutions it is becoming clear that blood testing may have the limitation of procurement of serum/plasma on the field. This may be a lesser issue if saliva is used (58, 117, 129) as is the case for S100B. Since the Scandinavian guidelines integrate clinical findings with biomarker values, we believe that this should also be adopted for other biomarkers. In fact, as shown above, brain-derived biomarkers cannot be specific for a particular neurological disease: thus, clinical judgment synergistically aids interpretation of biomarker values.

Although S100B is incorporated into the Scandinavian guidelines as an option to reduce CT scanning, implementation of these guidelines has been difficult. One aspect seems to be the introduction of a new modality in these patients (a biomarker), although the poor specificity reasonably contributes to the implementation difficulties. Indeed, the diagnostic performance of S100B in mTBI is somewhat similar to the performance of D-dimer in pulmonary embolism (130), a blood test that has also been difficult to implement clinically. A better understanding of barriers to guideline implementation may facilitate future efforts.

As suggested in a stroke study (108) and by the original patent on S100B in brain diseases [see (131)], repeated monitoring of a biomarker may be a partial solution to the steep kinetic decay of the biomarker within the diagnostic window for mTBI. In the future, monitoring prehospital biomarker trends compared to single-point measurement will be needed if the biomarkers are to be more broadly applied to clinical practice.

In conclusion, blood (or saliva) neurobiomarkers are reaching maturity at least in the TBI space. The future shall bring new discoveries and refinement of use, as in the case of GFAP and S100B. These show similarities and differences; the latter perhaps should be further explored to develop a combination test that exploits the strengths and lessens the weaknesses of these two popular means to diagnose TBI.

## Data Availability Statement

The original contributions presented in the study are included in the article/supplementary material, further inquiries can be directed to the corresponding author/s.

## Author Contributions

DJ, SM, JP, and JU each contributed to the different sections of this manuscript. DJ coordinated this international effort. All authors contributed equally to the design of this article. All authors contributed to the article and approved the submitted version.

## Funding

JP has received funding from the Academy of Finland (#17379), Competitive State Research Financing of the Expert Responsibility area of Turku University Hospital, Finland (#11129), and the Maire Taponen Foundation. SM has received funding from the Italian Ministry of Health (grant # GR-2013-02354960).

## Conflict of Interest

DJ owns shares in FloTBI, a start-up company specializing in salivary biomarkers of brain health. The remaining authors declare that the research was conducted in the absence of any commercial or financial relationships that could be construed as a potential conflict of interest.

## Publisher's Note

All claims expressed in this article are solely those of the authors and do not necessarily represent those of their affiliated organizations, or those of the publisher, the editors and the reviewers. Any product that may be evaluated in this article, or claim that may be made by its manufacturer, is not guaranteed or endorsed by the publisher.
